# A Maximal Correlation Framework for Fair Machine Learning

**DOI:** 10.3390/e24040461

**Published:** 2022-03-26

**Authors:** Joshua Lee, Yuheng Bu, Prasanna Sattigeri, Rameswar Panda, Gregory W. Wornell, Leonid Karlinsky, Rogerio Schmidt Feris

**Affiliations:** 1 Department of Electrical Engineering and Computer Science, Massachusetts Institute of Technology, Cambridge, MA 02139, USA; jk_lee@mit.edu (J.L.); gww@mit.edu (G.W.W.); 2 MIT-IBM Watson AI Lab, IBM Research, Cambridge, MA 02139, USA; psattig@us.ibm.com (P.S.); rpanda@ibm.com (R.P.); leonidka@il.ibm.com (L.K.); rsferis@us.ibm.com (R.S.F.)

**Keywords:** fairness, HGR maximal correlation, independence criterion, separation criterion

## Abstract

As machine learning algorithms grow in popularity and diversify to many industries, ethical and legal concerns regarding their fairness have become increasingly relevant. We explore the problem of algorithmic fairness, taking an information–theoretic view. The maximal correlation framework is introduced for expressing fairness constraints and is shown to be capable of being used to derive regularizers that enforce independence and separation-based fairness criteria, which admit optimization algorithms for both discrete and continuous variables that are more computationally efficient than existing algorithms. We show that these algorithms provide smooth performance–fairness tradeoff curves and perform competitively with state-of-the-art methods on both discrete datasets (COMPAS, Adult) and continuous datasets (Communities and Crimes).

## 1. Introduction

The use of machine learning in many industries has raised many ethical and legal concerns, especially that of fairness and bias in predictions, e.g., [1,2]. As systems are trusted to aid or make decisions regarding loan applications, criminal sentencing, and even health care, it is vital that unfair biases do not influence them.

However, mitigating these biases is complicated by ever-changing perspectives on fairness, and a good system for enforcing fairness must be adaptable to new settings. In particular, there are often competing notions on fairness. Two of these popular notions are independence and separation (a third condition, sufficiency, is beyond the scope of this paper), as discussed in [3]. Independence ensures that predictions are independent from membership in a protected class, so that one achieves equal favorable outcome rates across all groups, and it arises in applications such as affirmative action [4]. Separation is designed to achieve equal type I/II error rates across all groups by enforcing independence between predictions and membership in a protected class conditional on the class label. This criterion is used to measure fairness in recidivism predictions and bank loan applications. A significant body of work, including [3,5,6,7], has gone into explaining that independence and separation are inherently incompatible for non-trivial cases, and their applicability needs to be determined by the application and the stakeholders. This motivates us to construct a framework that is flexible enough to handle different fairness criteria and to do it with different modalities of data (discrete vs. continuous data, for example).

This bias mitigation must also be balanced out with the system’s usefulness, and often, one must tune the tradeoff between the fairness (as measured in the particular context) and performance according to a current situation, which can be a difficult process if the tradeoff curve is not smooth. Generating the frontier of possible values can be computationally infeasible or impossible if the algorithm does not have a regularization parameter to adjust (see, [8,9]), thus making it difficult to achieve this balance, which makes the fast generation of fair classifiers even more important.

Different contexts also require different points of intervention during the learning process to ensure fairness. *Pre-processing* approaches ([8,10,11,12,13,14]) modify the data to eliminate bias, whereas *post-processing* approaches ([15,16,17,18]) modify learned features/predictions from existing models to be more fair. We focus on the *in-processing* approach [9,19,20,21], where the fairness criteria are directly incorporated in the training objective to produce fairer learned features. Motivated by few-shot applications where only a pre-trained network and few samples labeled with the sensitive attribute are available, we also seek a method that is applicable in a post-processing manner when we have access to only a small number of samples labeled with the sensitive attribute that we wish to be fair about, which would arise in settings where collecting this information can be very difficult.

In this paper, we frame the ideas of independence and separation in a way that allows a relevant regularizer or penalty term to be derived in addition to a measure of fairness, which is useful in enforcing fairness while also tractable, admitting an optimization algorithm (e.g., if used as an objective for a neural net trained using gradient descent, it must be differentiable), and easily computed. Existing approaches can struggle with efficiency, can fail to provide good control over the performance–fairness tradeoff, and/or can only deal with either discrete or continuous data.

We make the following contributions in this paper:

- We present a universal framework justified by an information–theoretic view that can inherently handle the popular fairness criteria, namely independence and separation, while seamlessly adopting both discrete and continuous cases, which uses the maximal correlation to construct measures of fairness associated with different criteria; then, we use these measures to further develop fair learning algorithms in a fast, efficient, and effective manner.
- We show empirically that these algorithms can provide the desired smooth tradeoff curve between the performance and the measures of fairness on several standard datasets (COMPAS, Adult, and Communities and Crimes), so that a desired level of fairness can be achieved.
- Finally, we perform experiments to illustrate that our algorithms can be used to impose fairness on a model originally trained without any fairness constraint in the few-shot regime, which further demonstrates the versatility of our algorithms in a post-processing setup.

## 2. Background

### 2.1. Fairness Objectives in Machine Learning

Consider the standard supervised learning scenario where we predict the value of a target variable Y∈Y using a set of decision or predictive variables X∈X with training samples $\left\{\left(x_{1},y_{1}\right),\ldots ,\left(x_{n},y_{n}\right)\right\}$. For example, *X* may be information about an individual’s credit history, and *Y* is whether the individual will pay back a certain loan. In general, we wish to find features f(x), which are predictive of *Y*, so that we can construct a good predictor $\hat{y}=T\left(f\left(x\right)\right)$ of *y* under some loss criteria $L(\hat{y},y)$.

Now, suppose we have some sensitive attributes D∈D we wish to be “fair” about (e.g., race, gender), and training samples $\left\{\left(x_{1},y_{1},d_{1}\right),\ldots ,\left(x_{n},y_{n},d_{n}\right)\right\}$. For example, in the criminal justice system, predictions about the chance of recidivism of a convicted criminal (*Y*) given factors such as the nature of the crime and the number of prior arrests (*X*) should not be determined by race (*D*). This is a known issue with the COMPAS recidivism score, which, despite not using race as an input to make decisions, still leads to systematic bias toward members of certain races in the output score as in [22,23].

The two most popular criteria for fairness are independence and separation. Independence states that for a feature to be fair, it must satisfy the independence property $\hat{Y}⊥D$ or f(x)⊥D. The intuition is simple: if the prediction/feature is independent of the sensitive attribute, then no information about the sensitive attribute is used to predict *Y*. This criterion has been studied under the lens of *demographic parity* and *disparate impact* in [3], and it admits a class of fairness measures based on the degree of dependence between f(X) and *D*. For example, independence is satisfied if and only if the mutual information I(f(X);D) is zero. When *D* is binary, another popular class of measures used by the US Equal Employment Opportunity Commission [4] is the disparate impact, which is defined as $D\left(P(Y|D=1);P(Y|D=0)\right)=\frac{P(\hat{Y}=1|D=0)}{P(\hat{Y}=1|D=1)}$.

Separation requires the conditional independence property $(\hat{Y}⊥D)|Y$ or (f(X)⊥D)|Y. This criterion allows for a violation of demographic parity to the extent that it is justified by the target variable. In the general case, this criterion suggests a fairness measure based on the conditional dependence between $\hat{Y}$ and *D* conditioned on *Y*. In the case where *D* is binary, we obtain the *equalized opportunities* (EO) measures in [3], which are given by the differences in error rates for the two groups (e.g., the difference between the false positive rates for D=0,1). For a more complete discussion of the advantages and disadvantages of these two criteria, please refer to [3].

### 2.2. Maximal Correlation

Since these fairness criteria are expressed as enforcing independencies with respect to joint distributions, we look for constraints that reduce the dependency between variables. In particular, the right formulation of correlation between learned features and sensitive attributes can provide a framework for measuring and optimizing for fairness. One effective measure applicable to both continuous and discrete data is the Hirschfeld–Gebelein–Renyi (HGR) maximal correlation, which is a measure of nonlinear correlation that originated in [24] and is further developed in [25,26]. The HGR maximal correlation between two random variables is equal to zero if and only if the two variables are independent, and it increases in value the more correlated they are (i.e., the more biased/unfair).

**Definition** **1.** *For two jointly distributed random variables X∈X and Y∈Y, given 1≤k≤K−1 with K=min{|X|,|Y|}, the HGR maximal correlation problem is*

(1) $$\left(f^{*},g^{*}\right)≜\;\underset{\begin{array}{c} f:X\to R^{k},\;g:Y\to R^{k} \end{array}}{arg max}\;E\left[f^{T}\left(X\right)\;g\left(Y\right)\right],$$

*with constraints*

(2) $$\begin{array}{cc} E\left[f(X)\right] & =E\left[g(Y)\right]=0,\;E\left[f\left(X\right)f^{T}\left(X\right)\right]=E\left[g\left(Y\right)g^{T}\left(Y\right)\right]=I, \end{array}$$

and expectations taken over $P_{X,Y}$. We refer to $f^{*}$ and $g^{*}$ as maximal correlation functions, with $f^{*}={\left(f_{1}^{*},\ldots ,f_{k}^{*}\right)}^{T}$ and $g^{*}={\left(g_{1}^{*},\ldots ,g_{k}^{*}\right)}^{T}$, and the associated maximal correlations are

(3) $$\sigma \left(f_{i}^{*}\;g_{i}^{*}\right)≜E\left[f_{i}^{*}\left(X\right)\;g_{i}^{*}\left(Y\right)\right],\;for\;\;i=1,\ldots ,k,$$

*and the HGR maximal correlation is*

(4) $${\mathrm{HGR}}_{k}\left(X,Y\right)≜E\left[f^{*T}\left(X\right)\;g^{*}\left(Y\right)\right]=\sum_{i=1}^{k}\sigma \left(f_{i}^{*}\;g_{i}^{*}\right).$$

Note that the original definition of HGR maximal correlation is the special case of our definition when k=1 (see, [27]). This generalization of maximal correlation analysis enables us to produce more than one feature mapping by solving the maximal correlation problem, and these feature mappings can be used in other applications, including ensemble learning, multi-task learning, and transfer learning [28,29].

### 2.3. Related Work

Independence and separation have been studied in many works. Most existing approaches fail to provide an efficient solution in both discrete/continuous settings. Ref. [11] develops an optimizer using absolute difference in odds $|P\left(\hat{Y}=1|D=1\right)-P\left(\hat{Y}=1|D=0\right)|$ as a regularizer, which requires discrete *Y* and *D* and was only applied to Naïve Bayes and Logistic Regression to enforce the independence criterion. In [16], a post-processing method is provided using a probabilistic combination of classifiers to achieve the desired ROC curves, which only applies when *D* is discrete. Alternatively, Ref. [8] proposes pre-processing the data beforehand to enforce fairness before learning, based on randomized mappings of the data subject to a fairness constraint defined by $J=\max(|\frac{P(\hat{Y}=1|D=1)}{P(\hat{Y}=1|D=0)}-1|,|\frac{P(\hat{Y}=1|D=0)}{P(\hat{Y}=1|D=1)}-1|)$. Again, this method is only designed for independence with discrete *Y* and *D*, and it requires processing the entire dataset, which is computationally complex. Ref. [30] propose the use of a robust log-loss predictor for fairness, but in practice, it requires that *Y* be discrete.

Other methods can also be limited in their ability to handle all dependencies between variables. Ref. [31] uses a covariance-based constraint to enforce fairness, so it likely would not do well on other metrics. Furthermore, it is strictly a linear penalty rather than our non-linear formulation and penalizes the predictions of the system rather than the features learned. This limits the relationships between variables it can capture. An adversarial method is proposed in [20] to enforce independence or separation, but it requires the training of an adversary to predict the sensitive attribute, which can introduce issues of convergence and bias.

Recently, Ref. [9] propose the use of the HGR maximal correlation as a regularizer for either the independence or the separation constraint. In contrast to our approach dealing with the maximal correlation directly, they use a $\chi^{2}$ divergence computed over a mesh grid to upper bound the HGR maximal correlation during the optimization of the classifier (either a linear regressor or a Deep Neural Net (DNN)). This method applies to cases where *X* is continuous and *Y* and *D* are either continuous or discrete variables, but it scales poorly with the bandwidth and dimensionality of *D*, and it treats the discrete case in the same way as the continuous case, resulting in slow performance on discrete datasets.

There are other works that use either an HGR-based or mutual information-based formulation of fairness but do not generalize to more than one setting. Refs. [32,33] use correlation-based regularizers but can only be used in the independence case. Furthermore, Ref. [33] only works with discrete targets, and only uses a single mode of the HGR maximal correlation (as opposed to multiple modes, which our method makes use of) for regularization, which limits the information it can encapsulate, and it is also not designed for continuous sensitive attributes. Ref. [34] also develops a method that can only be used for independence, and it requires training an additional network in order to evaluate a bound for the mutual information which can be used to as a fairness penalty, thus increasing the complexity and required runtime. Finally, Ref. [35] approximates the mutual information with a variational formulation, but it does not include a formulation for continuous labels.

## 3. Maximal Correlation for Fairness

Equipped with the HGR maximal correlation as a measure of dependence, we explore its use as a fairness penalty. Depending on the data modality (discrete/continuous) and the fairness criteria (independence/separation), the resulting fair learning algorithm takes different specifically tailored forms. In this section, we demonstrate how to derive these regularizers and algorithms to ensure the aforementioned fairness objectives for both discrete and continuous cases.

### 3.1. Maximal Correlation for Discrete Learning

In this subsection, the decision variable *X*, target variable *Y*, and sensitive attribute *D* are discrete random variables defined on alphabets X, Y, and D, respectively.

We first describe how to solve the discrete maximal correlation problem using a divergence transfer matrix (DTM)-based approach. As it is shown later, it is more convenient to work with their equivalent representation via DTM instead of the joint distribution $P_{X,Y}$.

**Definition** **2.** *The divergence transfer matrix (DTM) $B_{Y,X}\in R^{\left|Y|\times |X\right|}$ associated with joint distribution $P_{X,Y}$ is given by*

(5) $$B_{X,Y}\left(x,y\right)≜\frac{P_{X,Y}\left(x,y\right)}{\sqrt{P_{X}\left(x\right)}\sqrt{P_{Y}\left(y\right)}}.$$

The following useful result expresses that the maximal correlation problem can be solved by simply computing the singular value decomposition (SVD) of the DTM B in the discrete case.

**Theorem** **1** ([27]). *Assume that the SVD of DTM $B_{Y,X}$ takes the form*

(6) $$B_{Y,X}=\sum_{i=0}^{K-1}\sigma_{i}\psi_{i}^{Y}{\left(\psi_{i}^{X}\right)}^{T},$$

*with singular values $\sigma_{0}\geq \sigma_{1}\geq \cdots \geq \sigma_{K-1}$, singular vectors $\psi_{i}^{Y}$, $\psi_{i}^{X}$, and K=min{|X|,|Y|}. Then, we have*

(7) $$\sigma_{0}=1,\;\psi_{0}^{X}\left(x\right)=\sqrt{P_{X}\left(x\right)},\;\psi_{0}^{Y}\left(y\right)=\sqrt{P_{Y}\left(y\right)},$$

*and the maximal correlation functions are related to the singular vectors in the SVD:*

(8) $$f_{i}^{*}\left(x\right)=\frac{\psi_{i}^{X}\left(x\right)}{\sqrt{P_{X}\left(x\right)}},\;g_{i}^{*}\left(x\right)=\frac{\psi_{i}^{Y}\left(y\right)}{\sqrt{P_{Y}\left(y\right)}},$$

with associated maximal correlations $\sigma \left(f_{i}^{*}\;g_{i}^{*}\right)=\sigma_{i}$, for i=1,⋯,K−1. Thus, the conditional distribution $P_{Y|X}$ has the following decomposition:

(9) $$P_{Y|X}\left(y|x\right)=P_{Y}\left(y\right)\left[1+\sum_{i=1}^{K-1}\sigma_{i}f_{i}^{*}\left(x\right)g_{i}^{*}\left(y\right)\right].$$

As we can see from this theorem, the singular values $\sigma_{i}$ (since the associated maximal correlations is equal to the corresponding singular values of DTM, we abuse the notation a little bit and use σ to denote both of them) of the matrix $B_{Y,X}$ essentially characterize the dependence between two discrete random variables, and the singular vectors $\Phi^{X}=\left[\psi_{1}^{X},\cdots ,\psi_{k}^{X}\right]$ and $\Phi^{Y}=\left[\psi_{1}^{Y},\cdots ,\psi_{k}^{Y}\right]$ are equivalent to the maximal correlation functions f and g.

Since our goal is to construct feature mappings f(x) under fairness constraints, our algorithms in the discrete case are built on the following variational characterization of an SVD, which does not involve g(y):

**Lemma** **1** ([36]). *For any k≤K−1 and $\Phi_{X}\in R^{|X|\times \left(k+1\right)}$,*

(10) $$\max_{\Phi_{X}^{T}\Phi_{X}=I}{∥B\Phi_{X}∥}_{F}^{2}=\sum_{i=0}^{k}\sigma_{i}^{2},$$

*where ${∥A∥}_{F}≜\sqrt{\mathrm{tr}(A^{T}A)}$ denotes the Frobenius norm.*

#### 3.1.1. Independence

To ensure sufficient independence, we must construct feature mappings $f:X\to R^{k}$ so that the maximal correlations between f(X) and *Y* are large, while the ones between f(X) and *D* are small. Motivated by Lemma 1 and Theorem 1, we propose the following DTM-based approach to construct f:

(11) $$\max_{\Phi \in R^{\left|X|\times (k+1\right)}:\Phi^{T}\Phi =I}∥B_{Y,X}{\Phi ∥}_{F}^{2}-\lambda {∥B_{D,X}\Phi ∥}_{F}^{2},$$

where $B_{Y,X}$ and $B_{D,X}$ denote the DTMs of distribution $P_{Y,X}$ and $P_{D,X}$, respectively, and λ is the regularization coefficient that controls the penalty of the maximal correlations between f(X) and *D*. $\Phi^{*}=\left[\phi_{0}^{*},\phi_{1}^{*},\cdots ,\phi_{k}^{*}\right]$ is the solution of the optimization problem (11). As shown in Theorem 1, $B_{Y,X}$ and $B_{D,X}$ have a shared right singular vector $\sqrt{P_{X}\left(x\right)}$, and we can let $\phi_{0}^{*}=\sqrt{P_{X}\left(x\right)}$. Then, the feature mappings for independence can be obtained by normalizing other column vectors in $\Phi^{*}$

(12) $$f_{i}\left(x\right)=\phi_{i}^{*}\left(x\right)/\sqrt{P_{X}\left(x\right)},\;i=1,\cdots ,k.$$

We have the following remarks:

(1) The optimization problem in (11) can be written as $\max\mathrm{tr}(\Phi^{T}(B_{Y,X}^{T}B_{Y,X}-\lambda B_{D,X}^{T}$$B_{D,X})\Phi )$, and it can be solved exactly by computing the eigen decomposition of $B_{Y,X}^{T}B_{Y,X}-\lambda B_{D,X}^{T}B_{D,X}$.

(2) Lemma 1 states that the Frobenius norm squared $∥B_{Y,X}{F∥}_{F}^{2}$ corresponds to the squared sum of the singular values. Actually, the following lemma shows that $∥B_{Y,X}{F∥}_{F}^{2}$ can be further related to the mutual information I(X;Y) when the dependence between *X* and *Y* is weak.

**Lemma** **2** ([27]). *Let X∈X and Y∈Y be ϵ-dependent random variables; i.e., the $\chi^{2}$-divergence is bounded $D_{\chi^{2}}\left(P_{X,Y}∥P_{X}P_{Y}\right)\leq \epsilon$, then*

(13) $$I\left(X;Y\right)=\frac{1}{2}\sum_{i=1}^{K-1}\sigma_{i}^{2}+o\left(\epsilon^{2}\right).$$

(3) As suggested by Lemma 2, the optimization problem in (11) can also be interpreted as maximizing the mutual information between f(X) and *Y* while penalizing the mutual information I(f(X);D).

Once we solve (11) and obtain the feature mappings f(x), we can obtain the corresponding maximal correlation function g(y) for the target variable *Y* via one step of the alternating conditional expectations algorithm by [37]:

(14) $$g_{i}\left(y\right)\propto E_{p_{X|Y}\left(\cdot |y\right)}\left[f_{i}\left(X\right)\right],\;i=1,\ldots ,k.$$

In turn, g(y) can be computed by further normalizing the conditional expectations of f(X), so that the condition $E\left[g\left(Y\right)g^{T}\left(Y\right)\right]=I$ is satisfied. Finally, the predictions $\hat{Y}$ can be made following the Maximum A Posteriori (MAP) rule, where the posteriori distribution $P_{Y|X}\left(y|x\right)$ can be approximately computed by plugging the learned feature mappings f(X) and g(Y) into (9), i.e.,

(15) $$\hat{Y}=\underset{y\in Y}{arg max}P_{Y}\left(y\right)\left[1+\sum_{i=1}^{k}\sigma_{i}f_{i}\left(x\right)g_{i}\left(y\right)\right].$$

#### 3.1.2. Separation

For the separation criterion, we want to ensure sufficient conditional independence (f(X)⊥D)|Y. Here, we cannot simply replace the $B_{D,X}$ in (11) with a conditional DTM, as it involves three random variables and thus cannot be usefully expressed as a matrix. Since maximal correlation is related to mutual information as shown in Lemma 2, we consider the following formulation:

(16) $$\begin{array}{cc} \; & \max_{f}I\left(f\left(X\right);Y\right)-\lambda I\left(f\left(X\right);D,Y\right)\\ \; & =\max_{f}I\left(f\left(X\right);Y\right)-\lambda \left(I(f(X);Y)+I(f(X);D|Y)\right)\\ \; & =\max_{f}\left(1-\lambda \right)I\left(f\left(X\right);Y\right)-\lambda I\left(f\left(X\right);D|Y\right), \end{array}$$

where the first equality follows from the chain rule of mutual information and λ∈(0,1). Thus, we can control the conditional mutual information I(f(X);D|Y) by adding the joint mutual information I(f(X);D,Y) as a regularizer in the training process.

Note that Lemma 1 and Lemma 2 imply that mutual information can be approximated using DTM, as shown in (11) in an independence case. Accordingly, we approximate (16) using the following optimization problem to ensure the separation criterion for discrete data:

(17) $$\max_{\Phi \in R^{\left|X|\times (k+1\right)}:\Phi^{T}\Phi =I}∥B_{Y,X}{\Phi ∥}_{F}^{2}-\lambda {∥B_{D⊗Y,X}\Phi ∥}_{F}^{2},$$

where D⊗Y is the Cartesian product of *D* and *Y*, and $B_{D⊗Y,X}$ denotes the DTM of distribution $P_{D⊗Y,X}$. Once we obtained the solution $\Phi^{*}$, we could follow similar steps as in the independence case to get f(x) and g(y) and make predictions for the test samples.

### 3.2. Maximal Correlation for Continuous Learning

When *X*, *Y*, and *D* are all continuous and real-valued, computing the HGR maximal correlation becomes much more difficult, since the space of functions over real numbers is not tractable. Thus, we turn to approximations and begin by limiting our scope of learning algorithms to those that train models (e.g., neural nets) via gradient descent (or SGD) using samples, which encompasses most of the commonly used methods. Then, it follows that any approximation of the HGR maximal correlation used must be differentiable to calculate the gradient. Thus, we restrict the space of maximal correlation functions to be the family of functions that can be learned by neural nets, allowing us to compute the gradient while still providing a rich set of functions to search over.

#### 3.2.1. Independence

To ensure sufficient independence, we want to minimize the loss function $L(\hat{Y},Y)$ and the maximal correlation between f(X) and *D*. Then, our optimization (for a given λ) becomes:

(18) $$\min_{\begin{array}{c} f:X\to R^{m}\\ T:R^{m}\to Y \end{array}}L\left(T\left(f\left(X\right)\right),Y\right)+\lambda {\mathrm{HGR}}_{k}\left(f\left(X\right),D\right),$$

where ${\mathrm{HGR}}_{k}\left(f\left(X\right),D\right)=\max_{\begin{array}{c} g,\;h \end{array}}E\left[g^{T}\left(f\left(X\right)\right)\;h\left(D\right)\right]$, with $E\left[g(f(X))\right]=E\left[h(D)\right]=0$, and $E\left[g\left(f\left(X\right)\right)g^{T}\left(f\left(X\right)\right)\right]=E\left[h\left(D\right)h^{T}\left(D\right)\right]=I$. *m* is the dimension of the features f(X), *k* is the number of maximal correlation functions, and $g:R^{m}\to R^{k},\;h:D\to R^{k}$ are the maximal correlation functions relating f(X) with *D*. Given the difficulty of enforcing the orthogonalization constraint, we use a variational characterization of the HGR maximal correlation called Soft-HGR proposed in [29], which relaxes the orthogonal constraint:

(19) $${\mathrm{HGR}}_{\mathrm{soft}}\left(X,Y\right)≜\max_{\begin{array}{c} E\left[g(X)\right]=0\\ E\left[h(Y)\right]=0 \end{array}}\;E\left[g^{T}\left(X\right)\;h\left(Y\right)\right]\;-\frac{1}{2}\mathrm{tr}\left(\mathrm{cov}\left[g\left(X\right)\right]\mathrm{cov}\left[h\left(Y\right)\right]\right),$$

where cov[X] is the covariance matrix of *X*. [29] shows that this Soft-HGR formulation can be viewed as a low-rank approximation of the original HGR maximal correlation problem in the discrete case. Then, our learning objective becomes:

(20) $$\min_{\begin{array}{c} f:X\to R^{m}\\ T:R^{m}\to Y \end{array}}\max_{\begin{array}{c} g:R^{m}\to R^{k},\;h:D\to R^{k}\\ E\left[g(f(X))\right]=E\left[h(D)\right]=0 \end{array}}C,$$

where

$$\begin{array}{c} C=L\left(T\left(f\left(X\right)\right),Y\right)+\lambda E\left[g^{T}\left(f\left(X\right)\right)\;h\left(D\right)\right]-\frac{\lambda }{2}\mathrm{tr}\left(\mathrm{cov}[g(f(X))]\mathrm{cov}[h(D)]\right). \end{array}$$

We solve this optimization by alternating between optimizing f,T and optimizing g,h. In practice, we implement this by alternating between one step of gradient descent for f and *T* and five steps of gradient descent on g and h to allow the maximal correlation functions to adapt to the changing of features f.

#### 3.2.2. Separation

For separation, we use a similar argument as in the discrete case to ensure the conditional independence. Specifically, we solve the following optimization problem:

(21) $$\begin{array}{c} \min_{\begin{array}{c} f:X\to R^{m}\\ T:R^{m}\to Y \end{array}}L\left(T\left(f\left(X\right)\right),Y\right)+\lambda \left({\mathrm{HGR}}_{\mathrm{soft}}\left(f\left(X\right),D⊗Y\right)-{\mathrm{HGR}}_{\mathrm{soft}}\left(f\left(X\right),Y\right)\right). \end{array}$$

Note that for the first Soft-HGR term, we use g,h to denote the maximal correlation functions and $g^{\prime },h^{\prime }$ to denote the functions for the second term. Similar to the discrete case, the difference term allows us to approximate the conditional mutual information using two unconditional terms. Once again, we solve this optimization by alternating between optimizing f,T and optimizing $g,h,g^{\prime },h^{\prime }$.

#### 3.2.3. Few-Shot Learning

In the continuous case, our learning objective can also be applied a posteriori in a few-shot setting with a clasifier that has already been trained in a fairness-unaware manner on a large number of samples without the sensitive attribute label. In this case, we can formulate our objective as before and use the few samples containing the sensitive attribute to further train the network and force it to learn fairer features that are still predictive of the desired labels.

## 4. Experimental Results

In order to illustrate the effectiveness of our algorithms, we run experiments using the proposed algorithms on discrete (Adult and COMPAS) and continuous (Communities and Crimes) datasets.

### 4.1. Discrete Case

We test the proposed DTM-based approach on the ProPublica’s COMPAS recidivism dataset (https://github.com/propublica/compas-analysis (accessed on 14 February 2022)) and the UCI Adult dataset (https://archive.ics.uci.edu/ml/datasets/adult (accessed on 14 February 2022)), which were chosen as they contain categorical features and are used in prior works. More experiments for the discrete case can be found in the Appendix A.

For the COMPAS dataset, the goal is to predict whether the individual recidivated (re-offended) (*Y*) using the severity of charge, number of prior crimes, and age category as the decision variables (*X*). As discussed in [8], COMPAS scores are biased against African-Americans, so race is set to be the sensitive attribute (*D*) and filtered to contain only Caucasian and African-American individuals. As for the Adult dataset, the goal is to predict the binary indicator (*Y*) of whether the income of the individual is more than 50 K or not based on the following decision variables (*X*): age (quantized to decades) and education (in years), and the sensitive attribute (*D*) is the gender of the individual.

For both datasets, we randomly split all data into 80%/20% training/test samples. We first construct an estimate of DTM $\hat{B}$ with the empirical distribution of the training set; then, we solve the proposed optimization in (11) and (17) using $\hat{B}$ to obtain fair feature mappings $\hat{f}\left(x\right),\hat{g}\left(y\right)$. The predictions $\hat{Y}$ of the test samples $X^{\prime }$ are given by plugging the learned feature mappings $\hat{f}\left(x^{\prime }\right),\hat{g}\left(y\right)$ into the MAP rule (15), where $P_{Y}$ can be estimated from the empirical distribution ${\hat{P}}_{Y}$ on the training set.

For the independence case, we compare the tradeoff between the performance and the discrimination achieved by our method with that of the optimized pre-processing methods proposed in [8]. Note that we adopt the same settings as the experiments in [8] to do a fair comparison, and the reported results for their method are from their work. We plot the area under the ROC curve (AUC) of ${\hat{P}}_{Y|X^{\prime }}\left(y|x^{\prime }\right)$ compared to the true test labels $Y^{\prime }$ against the following standard discrimination measure derived from legal proceedings [4]:

(22) $$J=\max_{d,d^{\prime }\in D}|P_{\hat{Y}|D}\left(1|d\right)/P_{\hat{Y}|D}\left(1|d^{\prime }\right)-1|.$$

**Figure 1:**
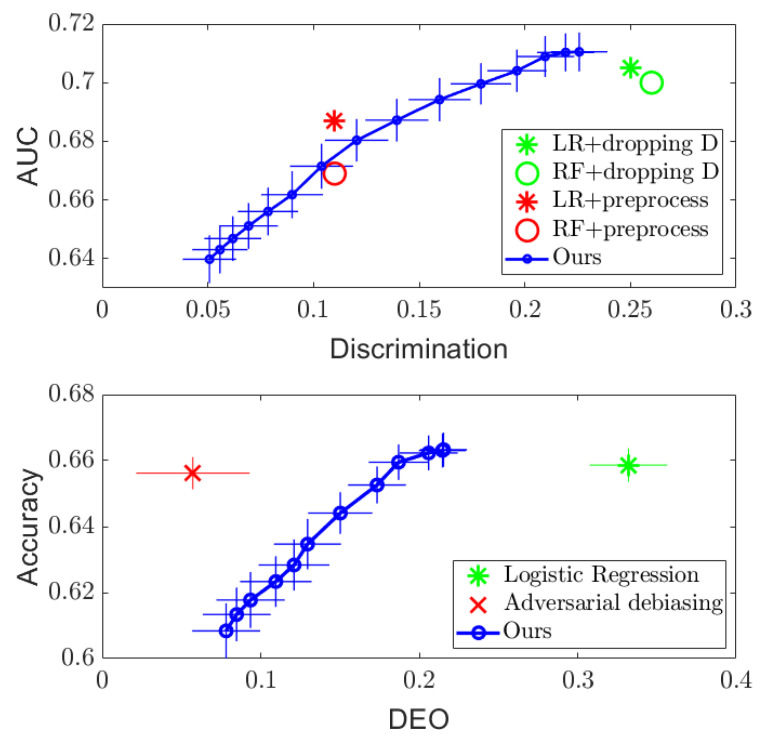
Regularization results on the COMPAS dataset, with AUC plotted against discrimination measure for independence (**Top**), and accuracy plotted against DEO for separation (**Bottom**), respectively.

**Figure 2:**
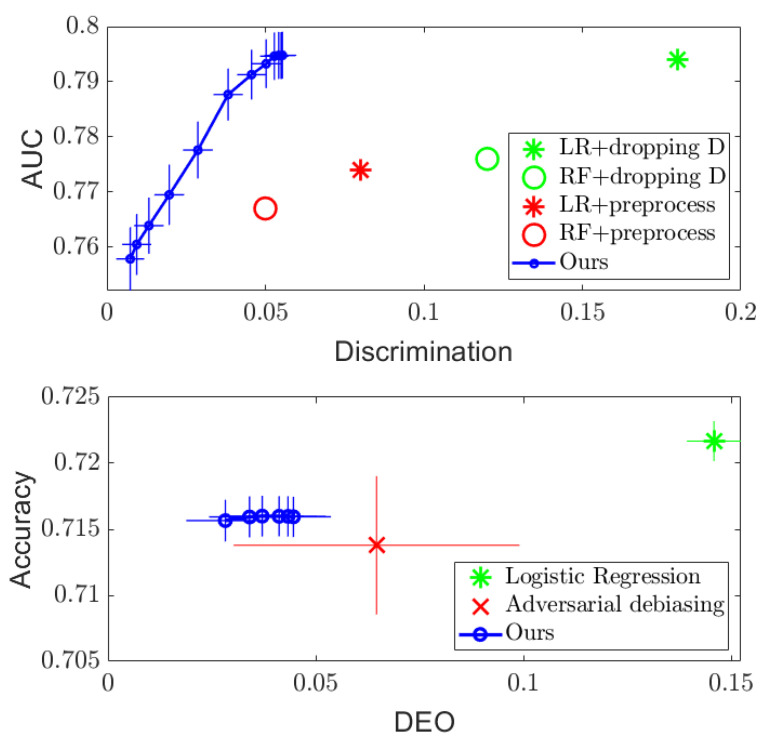
Regularization results on Adult dataset, with AUC plotted against discrimination measure for independence (**Top**), and accuracy plotted against DEO for separation (**Bottom**), respectively.

Figure 1 and Figure 2 (Top) show the results. For both datasets, it can be seen that simply dropping the sensitive attribute *D* and applying logistic regression (LR) and random forest (RF) algorithms cannot ensure independence between $\hat{Y}$ and *D*. However, the proposed DTM-based algorithm provides a tradeoff between performance and discrimination by varying the value of the regularizer λ in the optimization (11), which outperforms the optimized pre-processing methods in [8] on the Adult dataset and achieves similar performance on the COMPAS dataset. More importantly, the DTM-based algorithm provides a smooth tradeoff curve between the performance and discrimination, so that a desired level of fairness can be achieved by setting λ in practice. In addition, since our method only requires us to perform eigen-decomposition, it runs significantly faster than the optimized pre-processing method, which needs to solve a much more complex optimization problem. Empirically, we find at least a tenfold speed up in runtime compared to the existing methods.

For the separation criterion, we compare the balanced accuracy achieved by our algorithm with that of the adversarial debiasing method in [20] (implementation given in [2]) against the difference in equalized opportunities (DEO), which is another standard measure used commonly in the literature:

(23) $$\mathrm{DEO}=|P\left(\hat{Y}\;\;=\;\;1|D\;\;=\;\;1,Y\;\;=\;\;1\right)\;-\;P\left(\hat{Y}\;\;=\;\;1|D\;\;=\;\;0,Y\;\;=\;\;1\right)|.$$

The results on the COMPAS and Adult datasets are presented in Figure 1 and Figure 2 (Bottom). Compared to the naïve logistic regression, the proposed DTM-based algorithm dramatically decreases the DEO while maintaining similar accuracy performance on both datasets, which outperforms the adversarial debiasing method in [20] on the Adult dataset. We note that the accuracy and DEO curve achieved by the proposed algorithm in the separation setting has a smaller range compared to that in the independence setting. This is because the value of the regularizer λ is restricted in the separation optimization problem (17) to λ∈[0,1), but only to λ>0 for the optimization in (11). More details about the influence of the regularizer λ can be found in Appendix A.

### 4.2. Continuous Case

In the continuous case, we experiment on the Communities and Crimes (C&C) dataset (http://archive.ics.uci.edu/ml/datasets/communities+and+crime (accessed on 14 February 2022)). The goal is to predict the crime rate *Y* of a community given a set of 121 statistics *X* (distributions of income, age, urban/rural, etc.). The 122-th statistic (percentage of black people in the community) is used as the sensitive variable *D*. All variables in this dataset are real-valued. The dataset was split into 1794 training and 200 test samples. Following [9], we use a Neural Net with a 50-node hidden layer (which we denote as f(x)) and train a predictor $\hat{y}=T\left(f\left(x\right)\right)$ with the mean squared error (MSE) loss and the Soft-HGR penalty, varying λ. For Soft-HGR, we use two two-layer NNs with scalar outputs as the two maximal correlation functions g and h, and then, we trained them according to (20) (independence) or (21) (separation). Then, we computed the test MSE and test “discrimination” in each case.

**Figure 3:**
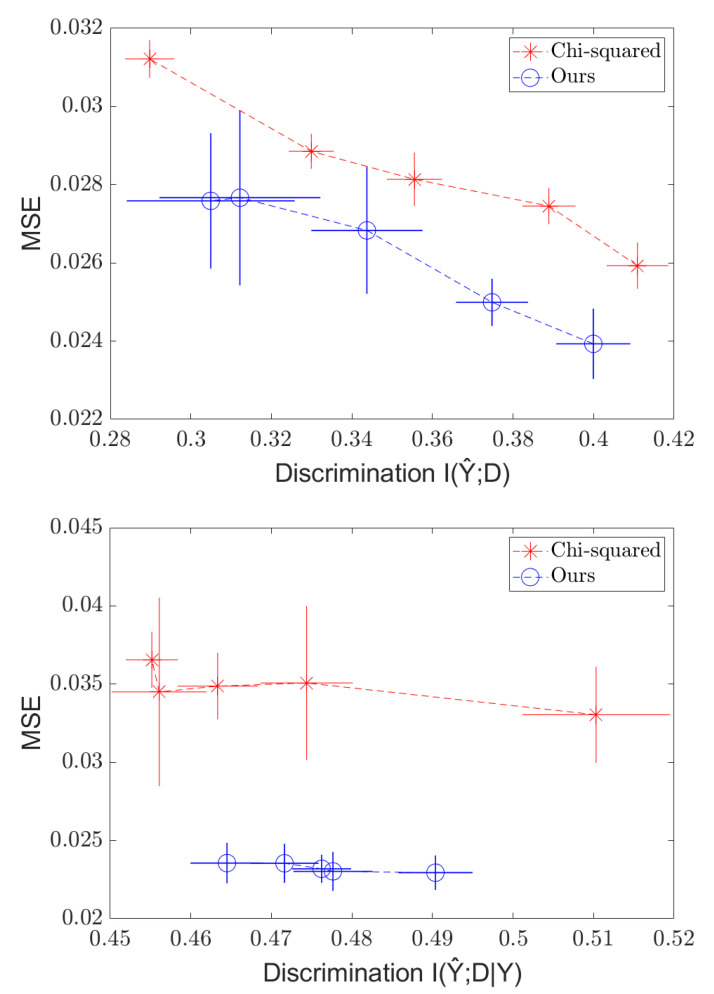
Independence (**top**) and Separation (**bottom**) regularization on the C&C dataset, with MSE plotted against $I(\hat{Y};D|Y)$.

For independence, our metric was $I(\hat{Y};D)$, which was approximated using a standard *k*NN-based mutual information estimator [38]. For separation, we computed $I(\hat{Y};D|Y)$ using the same estimator. We report the results of our experiment as well as that of the $\chi^{2}$ method of [9] with the same architecture. The results of the experiments are presented in Figure 3.

As expected, we see a tradeoff between the MSE and discrimination, creating a frontier of possible values. We also see that the Soft-HGR penalty provides modest gains compared to the $\chi^{2}$ method for both independence and separation.

Moreover, our method runs significantly faster than the $\chi^{2}$ method (on the order of seconds per iteration for our method versus just under a minute per iteration for the comparison method), as the $\chi^{2}$ method requires computation over a mesh grid of a Gaussian KDE, which scales with the product of the number of “bins” (mesh points) and the number of training samples, while our method only scales with the number of samples (O(n)), since it only requires passing over all the training samples a constant number of times per iteration. For large bandwidths, *d* can become quite large. KDE methods also scale poorly with dimensionality (see, [39]) in an exponential manner, and thus, if *d* is high-dimensional, the $\chi^{2}$ method would run much slower than our method, which can take in an arbitrarily-sized input and scale linearly with the dimensionality of the input multiplied by the number of samples. Empirically, we find that our method runs around five times faster.

**Figure 4:**
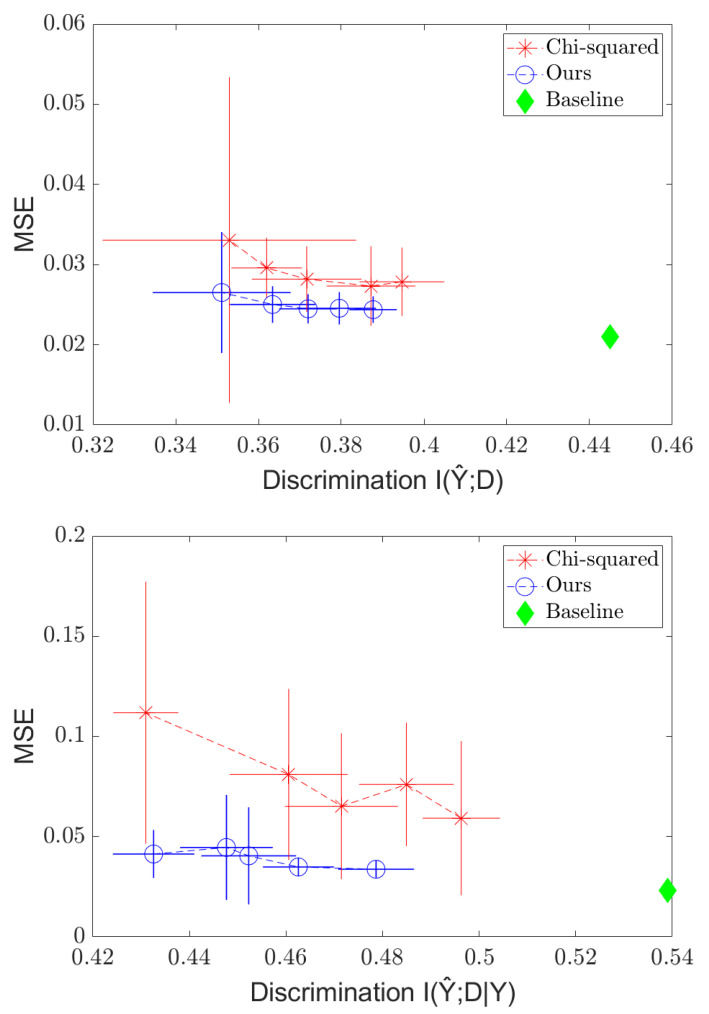
Independence (**top**) and Separation (**bottom**) regularization on the C&C dataset in the *few-shot* settings, with MSE plotted against $I(\hat{Y};D|Y)$.

We also run experiments to illustrate how our method’s simplicity allows it to adapt to the few-shot, few-epoch regime faster than that of the $\chi^{2}$ method. We take 10 “few-shot” samples from the training set; then, we train a network to predict *Y* from *X* without any fairness regularizer using the full training set. Then, we run five more iterations of gradient descent on the trained model using the fairness-regularized objective and the 10 few-shot samples, and we compare the separation results between the Soft-HGR and $\chi^{2}$ regularizer. We choose to compare to the $\chi^{2}$ regularizer as it is one of the few methods designed to handle continuous *D*. The results are shown in Figure 4. Once again, we see the tradeoff curve, and we see that our method outperform the $\chi^{2}$ method, and that it appears to be competitive with the standard case in just a few iterations, while the $\chi^{2}$ method is still far from achieving the original MSE. We also vastly outperform the baseline (before fairness regularization) model in reducing discrimination, at the cost of only a small increase in error. Thus, in situations where, due to ethical/legal issues, only a few samples labeled with the sensitive attribute can be collected, fairness can still be enforced.

## 5. Conclusions

As machine learning algorithms gain more relevance, more focus will be placed upon ensuring their fairness. We have presented a framework using the HGR maximal correlation, which provides effective and computationally efficient methods for enforcing independence and separation constraints, and derived algorithms for fair learning on discrete and continuous data, which provide competitive tradeoff curves. In addition, we have also shown promising results in the few-shot setting and suggested a method for rapidly adapting a classifier to improve fairness. In the future, it would be beneficial to extend this framework to other criteria (e.g., sufficiency) and to to determine how to use this framework to enforce fairness in a transfer learning setup coupled with the few-shot setting, to determine how to fairly adapt a classifier to a new task.

However, this method requires knowledge of the sensitive attribute for all samples during the training time, which can be impractical in some cases. Further extension into developing these regularizers with a limited number of such samples would be very useful.

## Figures and Tables

**Figure A1:**
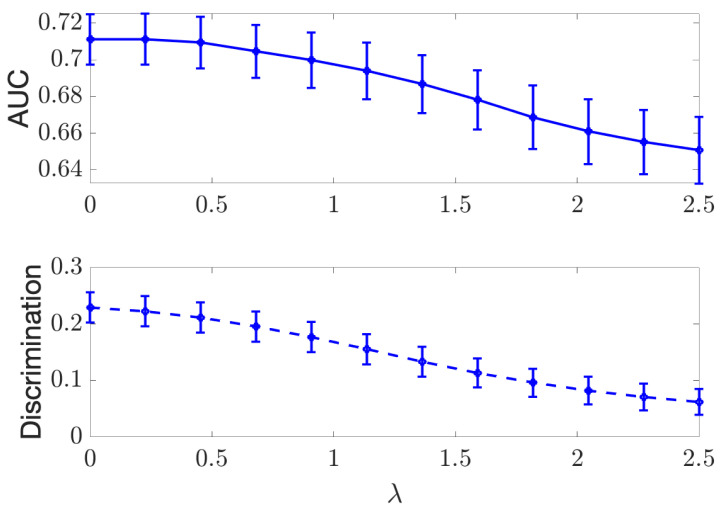
Results for independence regularization on the discrete COMPAS dataset, AUC results (**Top**) and discrimination measure *J* (**Bottom**) are plotted with respect to different values of λ.

**Figure A2:**
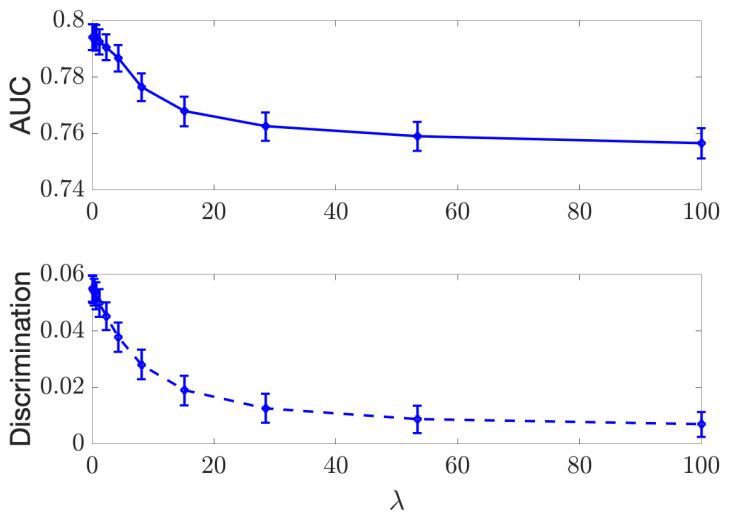
Results for independence regularization on the discrete Adult dataset, AUC results (**Top**) and discrimination measure *J* (**Bottom**) are plotted with respect to different values of λ.

**Figure A3:**
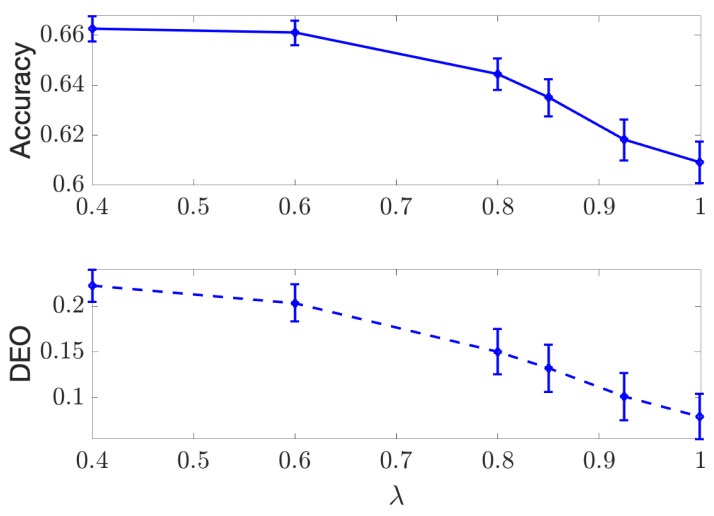
Results for separation regularization on the discrete COMPAS dataset, accuracy (**Top**) and DEO (**Bottom**) are plotted with respect to different values of λ.

**Figure A4:**
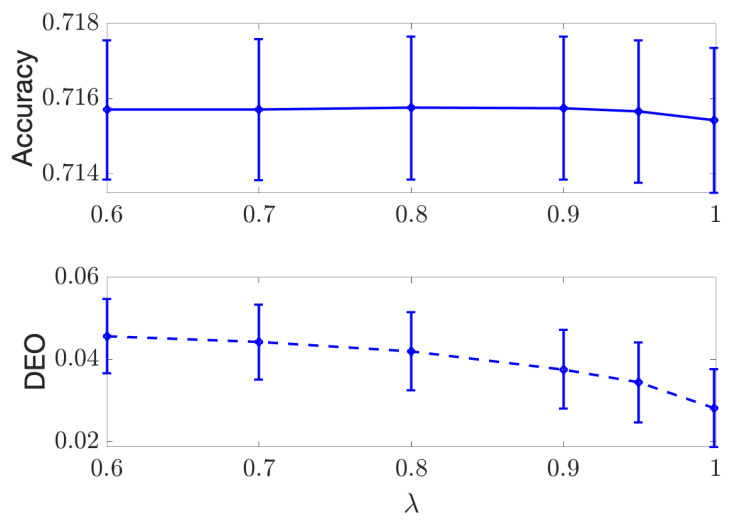
Results for separation regularization on the discrete Adult dataset, accuracy (**Top**) and DEO (**Bottom**) are plotted with respect to different values of λ.

## Data Availability

Data and code can be found in Section 4.

## Appendix A. Effect of the Regularizer in Discrete Case

In this section, we provide additional experiment results to demonstrate how the performance of classification and fairness measures change with different values of the regularizer.

We use the same setup described in Section 4.1 and present the results in Figure A1, Figure A2, Figure A3 and Figure A4. In Figure A1 and Figure A2, we plot the achieved AUC and Discrimination (measured with *J* in (21)) versus the value of λ for both COMPAS and Adult data using independence criterion. In Figure A3 and Figure A4, we plot the accuracy of the classifier and DEO versus λ for both datasets using separation criterion. As shown by all the figures, the performance of classification and fairness measures are all decreasing as we increase λ, and the proposed DTM-based algorithm is able to provide a smooth tradeoff curve between the performance and fairness measures.

Note that the value of the regularizer λ is restricted in the separation optimization problem to λ∈[0,1); therefore, the range of the achieved performance in Figure A3 and Figure A4 is smaller than that in Figure A1 and Figure A2.
